# CDSP: A Solution for Privacy and Security of Multimedia Information Processing in Industrial Big Data and Internet of Things

**DOI:** 10.3390/s19030556

**Published:** 2019-01-29

**Authors:** Xu Yang, Yumin Hou, Junping Ma, Hu He

**Affiliations:** 1 School of Computer Science and Technology, Beijing Institute of Technology, Beijing 100081, China; yangxu@tsinghua.edu.cn; 2 Institute of Microelectronics, Tsinghua University, Beijing 100084, China; hou-ym12@mails.tsinghua.edu.cn (Y.H.); mjpyd41@126.com (J.M.)

**Keywords:** privacy and security, internet of things, very long instruction word (VLIW), DSP, instruction set extension

## Abstract

With the widespread nature of wireless internet and internet of things, data have bloomed everywhere. Under the scenario of big data processing, privacy and security concerns become a very important consideration. This work focused on an approach to tackle the privacy and security issue of multimedia data/information in the internet of things domain. A solution based on Cryptographical Digital Signal Processor (CDSP), a Digital Signal Processor (DSP) based platform combined with dedicated instruction extension, has been proposed, to provide both programming flexibility and performance. We have evaluated CDSP, and the results show that the algorithms implemented on CDSP all have good performance. We have also taped out the platform designed for privacy and security concerns of multimedia transferring system based on CDSP. Using TSMC 55 nm technology, it could reach the speed of 360 MHz. Benefiting from its programmability, CDSP can be easily expanded to support more algorithms in this domain.

## 1. Introduction

We have entered the era of Big Data. The widespread nature of internet of things and wireless network, is making multimedia communication systems, such as on-line chatting, video conference and surveillance systems, becoming more and more popular. Since the process of multimedia communication systems involves data generation, storage, sending, receiving, sharing and so on, various security issues should be concerned. Data encryption algorithms could be adopted in multimedia communication systems to guarantee the security of data. Many kinds of data encryption algorithms have been released. This provides people with more options to choose according to their own needs. However, it also imposes a challenge for hardware design to have a large level of flexibility to adapt to different kinds of data encryption algorithms with limited time.

In this work, we focused on solving the privacy and security issue of multimedia surveillance system in internet of things domain, where high quality video and audio recorded by cameras needs to be compacted, encrypted and transferred to the control center through network, and be replayed in real-time on the monitors.

When evaluating the feature of the data stream of multimedia surveillance system, one finds that:

1. Data in the stream always contain private information, so encryption of the raw data is required to protect the data stream from disclosure before transferring. Thus data encryption algorithms need to be supported.
2. Since surveillance audio and video can be used as legal evidence to justify the fact, it is necessary to ensure the truth and reliability of the data stream. Hash functions and authentication could be of use for this.
3. Sometimes it is required to verify whether the audio and video are from a specific user. Thus digital signature should also be supported.
4. Requirement of real-time. Time delay from the scene to the control center should be less than 170 ms, which means that the work of video/audio encoding, encryption, decryption and video/audio decoding should be finished in 170 ms. According to the practical application, the peak throughput of the data stream should reach 150 Mbps.

Thus, the middleware platform of this kind of surveillance system should support a lot of different kinds of data encryption algorithms. In this work, we present our middleware solution based on CDSP, combining the programmability of DSP and the high efficiency of dedicated designed special operations. Benefiting from the programmability of DSP, new kinds of algorithms can be easily implemented, providing a high level of flexibility. Furthermore, the special operations designed dedicated for some algorithms can significantly reduce the code size, and largely enhance the performance. This is a new attempt in cryptographic DSPs, and the results show that our approach is both feasible and efficient.

The remainder of this paper is organized as follows: Section 2 will introduce the proposed DSP architecture. The design of dedicated special operations is discussed in Section 3. Related works are presented in Section 4. Section 5 gives the result of the evaluation. Finally, we give a conclusion in Section 6.

## 2. The Proposed CDSP Architecture

Our middleware solution is built based on a DSP called CDSP, which is designed as a 6-issue VLIW DSP. It provides high instruction level parallelism, and can greatly improve the performance on cryptographic algorithms execution. In this section we will introduce it in more detail.

### 2.1. Design of CDSP Core

**Figure 1:**
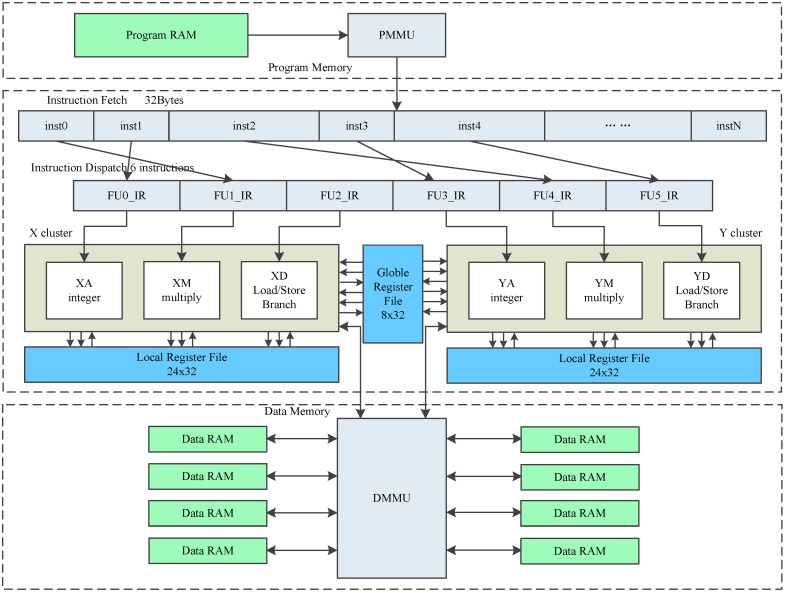
CDSP architecture.

CDSP core [1] is composed of four main parts: Memory, Instruction Fetch Unit (IFU), Instruction Dispatch Unit (IDU), and Execution Unit (EU). The architecture of CDSP is shown in Figure 1. CDSP adopts Harvard architecture and has separate Program Memory (PMEM) and Data Memory (DMEM). PMEM is 24 KB SRAM, with a 256-bit width port. PMEM is used to store CDSP program, and can be initialized using DMA. DMEM is 16 KB dual-port SRAM, and each port is 64-bit width. DMEM is used to store data stream, and the data stream is transferred through AXI bus under the control of DMA.

IFU reads instruction packets from PMEM. Each instruction packet is 256-bit width, containing eight to 16 instructions.

IDU seeks available instructions in the package and hands them out to EU. At most, six instructions can be dispatched each cycle.

EU is clustered, and the two clusters are named as X cluster and Y cluster. EU includes six function units, which are named as XA, YA, XM, YM, XD and YD separately. XA, XM and XD belong to X cluster. YA, YM and YD belong to Y cluster. XA and YA are arithmetic units, executing arithmetic and logic instructions, such as ADD, SUB, AND, XOR, ASL and LSR. XM and YM are multiplication units, executing multiplication instructions, and some arithmetic logic instructions. XD and YD are load/store units, loading data from DMEM to register, or storing data from register to DMEM. Some arithmetic logic instructions and branch instructions can also be executed in XD and YD units.

### 2.2. Design of Pipeline

**Figure 2:**
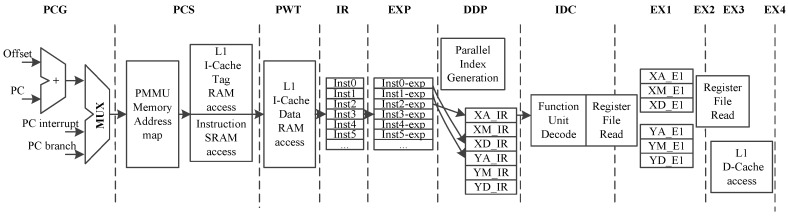
Design of CDSP pipeline.

CDSP includes 11 pipeline stages, as shown in Figure 2.

PCG (PC generation): This stage generates the next PC, which is chosen from the interrupt PC, branch PC and PC + 4.

PCS (PC send): This stage passes the generated PC to the next stage.

PWT (PC wait): This stage checks whether the instruction is valid in the instruction cache or not. If the instruction is invalid, instructions should be fetched from the PMEM.

IR (instruction return): We get the instructions in this stage.

EXP (instruction expansion): As the instructions are either 16-bit or 32-bit, we expand the instructions to the same length in this stage.

IDP (instruction dispatch): Instruction dispatch is completed in this stage. Since the function unit information is encoded in the instructions, we can get this information and dispatch the instructions to the corresponding function units. This procedure can be interpreted as predecode and dispatch.

IDC (instruction decode): This stage decodes the instructions.

EX1∼EX4 (instruction execution): Instructions are executed in this stage. Different instructions need 2∼4 stages to complete execution.

### 2.3. Design of Register Files

CDSP has three register files, which are also clustered. Register files are also shown in Figure 1. X and Y cluster register files both contain 24 registers, which are named as X0∼X23 and Y0∼Y23 separately. There is a global register file G containing eight registers named as G0∼G7. All the registers are 32-bit width. X unit instructions can use X cluster registers and G registers, and Y unit instructions can use Y cluster registers and G registers. Two adjacent registers can be used as a register pair, forming a bigger operand. For example, X1:X0 is a register pair and forms a 64-bit operand.

X and Y are two symmetrical clusters. Taking advantage of the symmetrical architecture, CDSP can access 56 registers using only 5 bits of the instruction. Usually, 5 bits can only address 32 registers. A large number of registers can help exploit Instruction Level Parallelism (ILP) and increase the performance. Some algorithms can also take advantage of the symmetrical architecture to run in parallel.

### 2.4. Design of General Instructions

CDSP is a RISC processor, and its Instruction Set consists of both general instructions and dedicated designed special instructions. General instructions include arithmetic and logic instructions, shift instructions, multiplication instructions, load instructions and branch instructions. Dedicated instructions are designed for specific cryptographic algorithms in order to improve the performance. General instructions will be described in this part.

Arithmetic instructions: Addition (ADD/ADC) and subtraction (SUB/SUBB). The operands could be 32-bit signed or unsigned numbers, and the operation could be addition with carry or subtraction with borrow.

Logic instructions: Bitwise AND, bitwise OR, bitwise XOR and bitwise Negate (NOT). Operands can be immediate data or come from registers.

Move instructions: Move a data from one register to another (MOV).

Shift instructions: Arithmetic Shift Right (ASR), Logic Shift Right (LSR), Arithmetic Shift Left (ASL), and Barrel Rotate Left (ROL).

Multiplication instructions: Multiplication (MUL) and Accumulation (ACC). Multiplication instructions occupy two pipeline stages.

Pack instructions: Pack instructions switch the byte sequence in a 32-bit operand, or select some bytes from two operands and build up a new word.

LOAD/STORE instructions: Load (LD) data from data memory to registers, or store (ST) data from register to data memory. A RISC processor always uses powerful load-store instructions to finish data transfer between registers and memory. CDSP LD instructions use base register and offset register to calculate the memory address, and the base register can increase or decrease automatically.

**Branch instructions:** Branch to the target PC and flush the instructions in pipeline (B); Branch to the target PC without flushing the instructions in pipeline (BD); Call subroutine (CALL); Return from subroutine (RET).

### 2.5. Design of Interfaces

**Figure 3:**
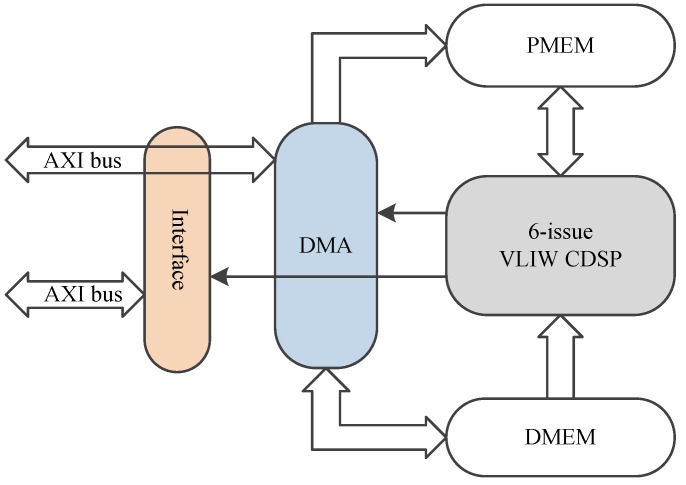
Architecture of CDSP system.

The architecture of CDSP system is shown in Figure 3. There is an AXI-lite slave interface in CDSP, which is used to control work flow, configure CDSP and check the status of CDSP. AXI is a widely used bus standard, making CDSP easy to integrate.

An embedded Direct Memory Access (DMA) with an AXI master interface is designed in CDSP, which is used to read and write data stream though AXI bus, with high bandwidth. The DMA can be controlled either by CDSP or AXI-lite.

## 3. Design of Dedicated Instructions

The CDSP platform we presented combines both the programmability of DSP and the high efficiency of dedicated designed special instruction to: (1) provide flexibility for implementing of different kinds of encryption algorithms, and (2) achieve high performance. Dedicated instruction design for cryptographic algorithms is an important feature of CDSP, and is also an innovation in cryptographic DSP design.

Many kinds of data encryption algorithms have already been implemented on our platform:

1. Block cipher algorithms [2,3,4,5], including DES, 3DES, AES and IDEA. We have designed dedicated instruction for AES and DES, while IDEA is implemented using only general instructions.
2. Hash [6] functions and public key algorithms. The study of public key algorithms are mainly focused on the ASIC approach in the literature. It is an innovation to realize these algorithms on a DSP platform. These algorithms are implemented using general instructions.

In this section, the design of dedicated instructions for DES and AES algorithms will be introduced.

### 3.1. Design for DES Algorithm

#### 3.1.1. Flow of DES Algorithm

**Figure 4:**
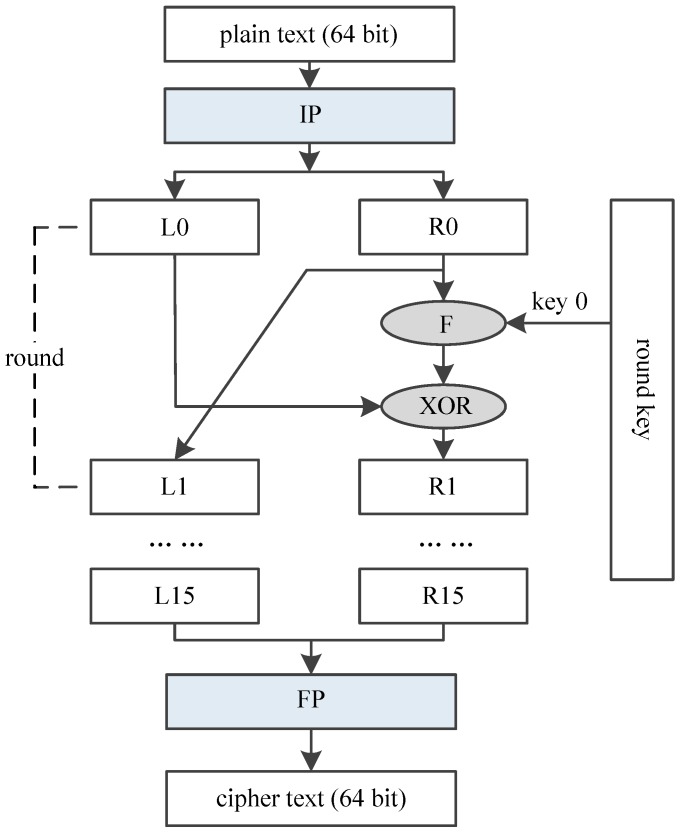
Architecture of DES.

DES encrypts 64-bit plain text, and generates a 64-bit cipher text, using a 64-bit key. DES algorithm could be divided into three function units as shown in Figure 4. The first part is an Initial Permutation (IP), which reorders the 64 bits of the plain text. The second part includes a Feistel (F) function and an operation of adding round key, which is iterated 16 times. The last part is a Final Permutation (FP), reordering the 64-bit intermediate data and outputting the final cipher text.

**Figure 5:**
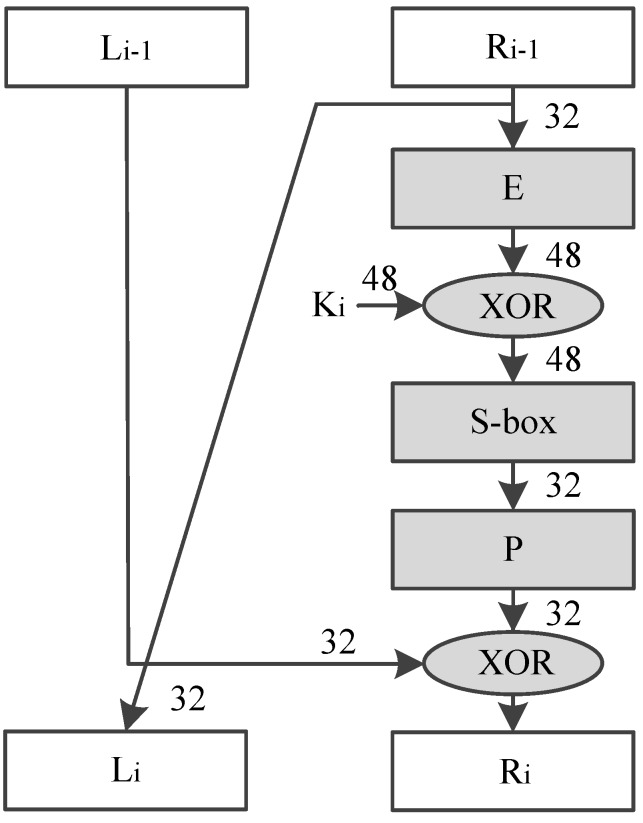
Data Encryption Standard (DES) round transformation.

Figure 5 shows the process of DES round transformation. 64-bit intermediate text is divided into 2 parts, named as $L_{i}$ and $R_{i}$ separately, both of which are 32-bit width. DES round transformation mainly contains 4 steps, and The 4 steps are the Feistel function (F):

(1) $R_{i}$ is transformed using a Expansion (E) permutation;
(2) Result of step (1) xor with round key ($K_{i}$);
(3) Result of step (2) is substituted using lookup table S-box;
(4) Result of step (3) is rearranged with a new Permutation (P).

#### 3.1.2. Dedicated Instruction Design for DES

If only general instructions are used to implement the DES algorithm, then a large number of instructions will be needed, considering the performing of the permutation, which would result in large code size. And also the performance might be low.

Three dedicated instructions are designed for the implementation of DES algorithm:

1. DESIP: Deigned to implement the IP operation. The source operand is a 64-bit data from a register pair, and the result is also 64-bit width and stored in a register pair.
2. DESFP: Deigned to implement the FP operation. Function of this instruction is the same as DSEIP.
3. DESRND: Designed to implement the DES round transformation. The source operand is a 64-bit intermediate result and a 48-bit round key. The output is a 64-bit intermediate result for the next round.

Table 1 shows the flow of DES algorithm with the help of dedicated designed special instructions. DES encryption can be finished in 20 cycles. Similarly, 3DES can be finished in 57 cycles. Since CDSP includes two clusters, two data blocks can be encrypted separately in X cluster and Y cluster in parallel, thus the performance will be doubled.

The comparison of code size is shown in Table 2. By the using of dedicated instructions, the code size can be more compacted, and the execution time also reduced.

### 3.2. Design for AES Algorithm

#### 3.2.1. Flow of AES Algorithm

**Figure 6:**
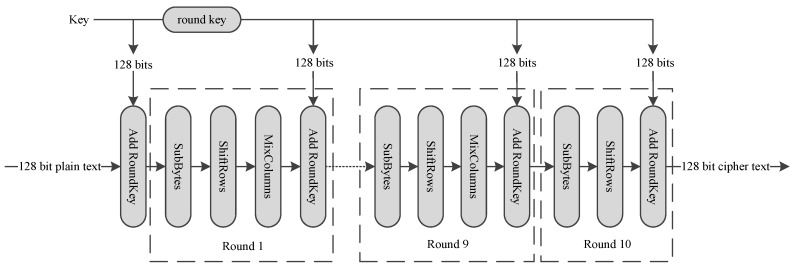
AES transformation.

**Figure 7:**
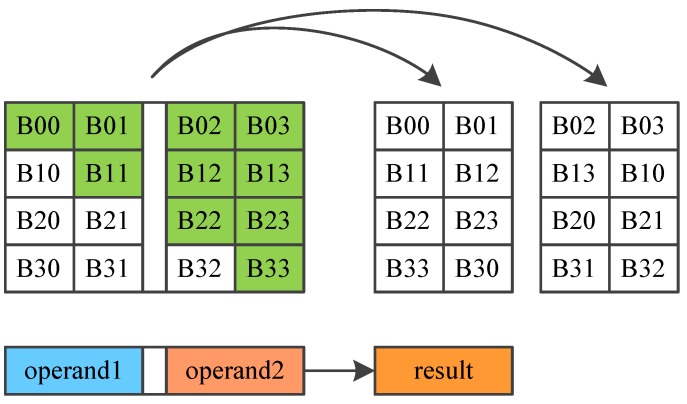
AES dedicated instructions AESSHF.

AES encrypts 128-bit plain text, and generates a 128-bit cipher text, using a 128-bit key. AES consists of 10 round transformations. The intermediate result between two rounds is a 16-byte data called mid-state, which is usually arranged in a 4 × 4 matrix. AES round transformation is based on this matrix. AES could be divided into four main operations as shown in Figure 6:

1. SubBytes: Each byte is used as an address to look up a SubBytes table and output a new byte, substituting for the initial byte.
2. ShiftRows: The last three rows of the matrix shift left cyclically for 1 byte, 2 bytes and 3 bytes separately, as shown in Figure 6.
3. MixColumns: Combines 4 bytes in each column. It can be implemented by using a fix value matrix to multiply the mid-state, as shown in Figure 7.
4. AddRoundKey: Round key is a 128-bit key expanded from the initial key. AddRoundKey conducts bitwise XOR operation between mid-state and the round key.

ShiftRows can be moved to the front of SubBytes, and the sequence will become ShiftRows→SubBytes→MixColumns→AddRoundKey.

#### 3.2.2. Dedicated Instruction Design for AES

Using general instructions only to implement SubBytes, ShiftRows and MixColumns operations will result in low throughput and large code size. Dedicated instructions are designed to enhance the performance of AES.

**Figure 8:**
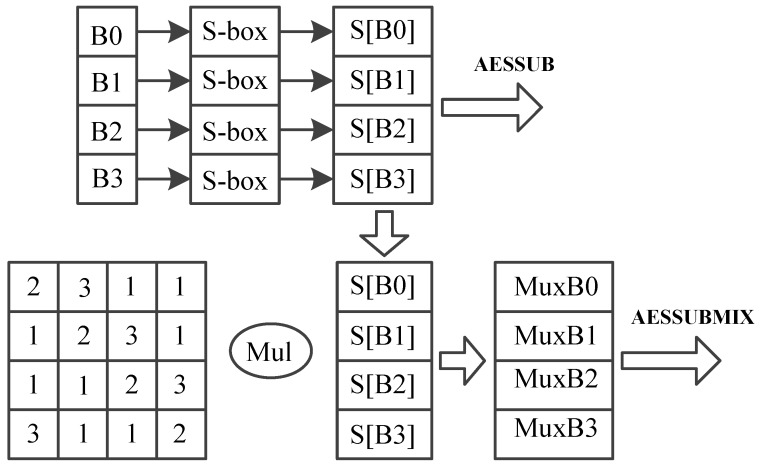
AES dedicated instructions AESSUB and AESSUBMIX.

Three dedicated instructions are designed for the implementation of DES algorithm:

1. AESSHF: AESSHF conducts ShiftRows, as shown in Figure 6. Each column of the matrix is stored in a register, and two registers make up a register pair, a register pair is used as an operand. AESSHF finishes the ShiftRows function using two source operands. The output is two new columns. AESSHF should run two times to finish the ShiftRows operation.
2. AESSUBMIX: AESSUBMIX conducts two operations, SubBytes and Mixcolumns, as shown in Figure 7. The instruction occupies two pipeline stages. At the first stage, 4 bytes are replaced according S-box table, and at the second stage, a fix-value matrix is multiplied by the S-box output, generating the result. Source operand is one column of the mid-state and the result is a column of the next mid-state.
3. AESSUB: AESSUB conducts SubBytes operation, as shown in Figure 8. AESSUB instruction outputs the S-box result and uses one pipeline stage. AESSUB instruction is used in the final round, because the final round dose not includes the MixColumns [7] operation.

Table 3 shows the flow of AES algorithm with the help of dedicated designed special instructions. One AES round can be finished in five clock cycles, shown as cycle 5 to 9. AES encryption can be finished in 59 cycles.

As shown in Table 4, code size can be noticeably reduced by using dedicated instructions.

## 4. Related Works

There is not much research reported on the implementation of cryptographic algorithms on a DSP platform combined with dedicated instruction extension, while some are similar to ours. We divide them into two categories.

### 4.1. Cryptographic Algorithms on a DSP

T. Wollinger et al. [8] research how well-suited high-end DSPs are for the AES algorithms. Five AES candidates: Mars, RC6, Rijndael, Serpent and Twofish are investigated and realized on a TMS320C6201 DSP. They optimize the C code to speed up the algorithm. They provide single-block and multi-block processing to enable the data blocks to be executed in parallel; this method is limited to be used under certain confidentiality modes.

TMS320C6201 DSP has 32 32-bit registers and eight independent functional units. The architecture of TMS320C6201 DSP is also divided into two parts and each part includes four functional units and 16 registers.

They compare the result with Pentium-Pro processor, both working under 200 MHz. It shows that the performance of TMS320C6201 is better than Pentium-Pro processor by about 32.3% on average.

K. Itoh et al. [9] also implement public-key cryptographic algorithms on TMS320C6201 DSP, including Rivest-Shamir-Adleman (RSA) [10,11,12], DSA and ECDSA [13]. The performances of RSA1024, DSA1024 and ECDSA160 achieve 11.7 ms, 14.5 ms and 3.97 ms respectively. The result is achieved mainly be optimization of modular multiplication and elliptic doubling operations. D. Xu et al. [14] realize AES algorithm on a configurable VLIW DSP called Jazz DSP. The computation units can be configured by software. They implement AES on three configurations and the best performance reaches 10.56 cycles/byte, in which case, eight VLIW slots are configured and two functions SubByte and Mixcolomn are converted to the designer defined computation unit to improve the performance.

Y. S. Zhang et al. [15] have designed a low-cost and confidentiality-preserving data acquisition framework for IoMT. They first used chaotic convolution and random subsampling to capture multiple image signals, assembled these sampled images into a big master image, and then encrypted this master image based on Arnold transform and single value diffusion. The encrypted image is delivered to cloud servers for storage and decryption service.

### 4.2. Instruction Set Extension for Cryptographic Algorithms

Intel proposes Advanced Encryption Standard (AES) new instruction set in 2010 [16]. The new instruction set includes six instructions designed for AES, of which four instructions realize AES encryption and decryption, and the other two support AES key expansion. The new instruction set makes AES simple to implement with small code size. The performance of AES with 128-bit key achieves 4.44, 4.56 and 4.49 cycle/byte under ECB, CBC and CTR mode respectively. The result comes from a processor based on Intel microarchitecture running at 2.67 GHz.

The IBM Power8 [17] processor also improves the performance on data encryption. It adds 11 new instructions to improve the performance of cryptographic algorithms, including AES, Galois Counter Mode (GCM) of operation for AES, SHA-2, and CRC. Vector and Scalar Unit (VSU) is also added to enhance the performance. Under CBC mode, the throughput of AES128 reaches about 680,000 KB/s with the processor running at 3.59 GHz.

Our implementation combines the features of these two approaches, which realizes cryptographic algorithms on a DSP with an extended instruction set.

## 5. Experimental Results

### 5.1. Experimental Framework

Function simulation of CDSP is achieved by Synopsis VCS and Verdi joint simulation, working under Redhat system. FPGA verification is conducted on XC5VLX330T from Xilinx vertex 5 series. Verification is based on Synopsis VMM (Verification Methodology Manual), using system Verilog. Using VMM, we can generate random test cases or test cases with constraints.

C language is used to build a C-model for certain cryptographic algorithm. Furthermore, we use C-model to generate the correct result, called the golden result.

The programs implementing different cryptographic algorithms are written in CDSP assembly language. The assembler of CDSP is designed based on GNU Binutils binary tool set. We compiled the assembly programs, sent the firmware, together with the test cases, to the DSP, and got the simulation result. The result will be compared with the golden result, and whether the result is correct will be reported.

### 5.2. Results

#### 5.2.1. Results for Block Cipher Programs

Table 5 lists the performance of DES, 3DES, AES and IDEA in CDSP. The clock cycles and throughput for one block encryption are given, when CDSP is running at 360 MHz. The 3rd column gives the performance with no encryption mode. The following columns give the performance in different confidentiality modes, including ECB, CBC, CFB, OFB and CTR, which are defined in FIPS [18]. In CFB and OFB confidentiality modes, the block length is select as 64-bit for DES, 3DES, and IDEA, and 128-bit for AES. The programs running in CDSP can be easily modified to support more confidentiality modes.

Performance comparison of AES128 with general purpose processors is shown in Table 6. We compare the performance of our implementation with ARM7 and ARM9. The result shows that CDSP has dramatic advantage over the two ARM processors. In comparison, the architecture of our design provides higher level of parallelism and the dedicated instructions are useful to speed up CDSP.

We also compare the performance of CDSP with other approaches of ISA extension, as shown in Table 7. Power8 processor adds 11 instruction to enhance the efficiency of AES. Intel processor adopts the AES new instruction set. The result shows that our implementation is better than Power8 in the performance of CPU cycles per Byte. According to Ref. [17], the throughput of Power8 for 128 bit encryption is about 680,000 KB/s, and the processor run under 3.59 GHz. We calculate that the performance of CPU cycles per Byte is 5.53. The result of Intel is based on Intel microarchitecture codename Westmere running at 2.67 GHz [16]. Our implementation shows close performance with Intel under CBC mode. Power8 and Intel processors are both ASIC. ASIC always has higher frequency than DSP. The parameter of CPU cycles per Byte is not affected by frequency and it provides a more fair comparison. The difference in the design of instructions and hardware results in different performance.

Table 8 shows the performance comparison of AES128 between CDSP and other DSP approaches. The result shows that CDSP has far better performance than Ref. [8,14]. DSPs always provide high level of parallelism, but the dedicated instructions make our design outweigh other DSPs in cryptographic algorithms.

#### 5.2.2. Results for Hash Function

Table 9 lists the performance of MD5 [20,21] and SHA-1 in CDSP. The second column lists the clock cycles consumed in one data block compression, which is 512-bit width. The third column shows the time consumed compressing one data block. The forth column gives the throughput when CDSP is running under 360 MHz. Since CDSP owns 6 function units, the arithmetic and logic operations in SHA-1 and MD5 can run in parallel. The VLIW architecture with 6-issue is good at exploiting instruction level parallelism and achieving better performance. CDSP can also implement other Hash functions through software development, such as SHA-256 and SHA-512.

#### 5.2.3. Results for Public Key Algorithms

Table 10 lists RSA and Elliptic Curve Cryptography (ECC) [22,23] performance when CDSP is running under 360 MHz. The first column shows the algorithms, including RSA using a 1024-bit key and a 2048-bit key, with and without applying CRT, and ECC using a 192-bit key and a 256-bit key. The second column lists the clock cycles used in Montgomery modular multiplication. The third column shows the number of multiplication operations used in these algorithms. The forth and the fifth columns list the clock cycles and total time consumed. The last column gives the number of executions of the algorithm per second.

In the third column, the number of multiplication operations are evaluated based on the assumption that half of the binary bits in a big number are 1, which is a worst case. Choice of the key and window width can affect the result significantly. In this paper, the window width is 2, and the performance can be raised by 8.7% on average compared with the performance when the window width is 1.

Table 11 shows that CDSP has equal RSA performance with Ref. [9]. Since both designs are DSPs and there are not dedicated instructions for RSA in our design, the result is understandable. In Ref. [9], the DSP works under 200 MHz, while our design works under higher frequency of 360 MHz.

In conclusion, CDSP shows satisfactory performance in cryptographic algorithms. Compared with other DSPs, CDSP shows far better performance for algorithms with dedicated instructions and close performance for algorithms without dedicated instructions. Compared with processor with dedicated cryptographic instructions, CDSP shows better performance than Power8 and close performance with Intel under CBC mode.

### 5.3. Silicon Implementation

**Figure 9:**
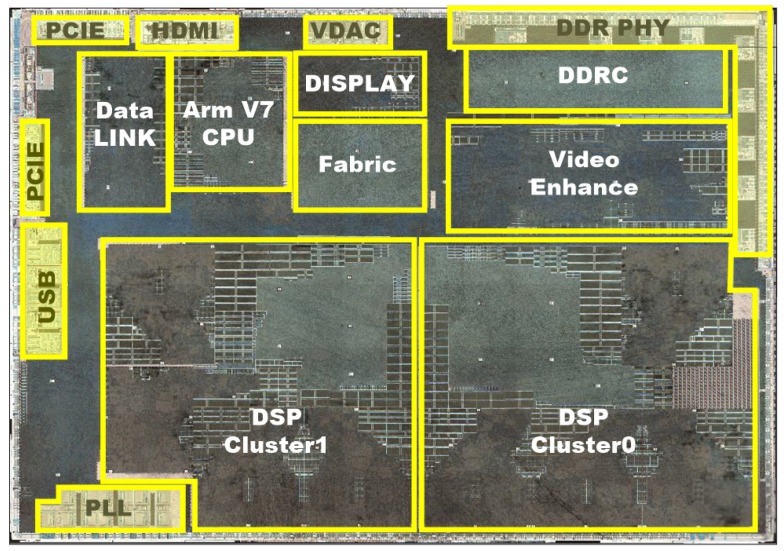
Layout of the multimedia surveillance system.

**Figure 10:**
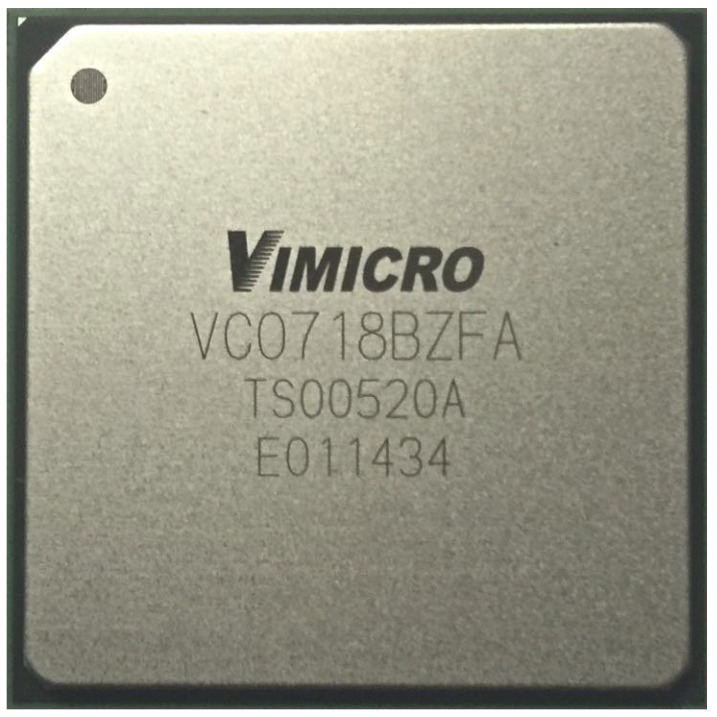
Photo of the chip.

We have implemented the multimedia surveillance system based on CDSP platform. The chip of the surveillance system is taped out and mass-produced. Using TSMC 55 nm technology, the synthesized frequency of CDSP achieves 360 MHz. The critical path is in the instruction dispatch stage. The dedicated instruction extension does not reduce the working frequency. The area is 186,000 gates and 40 KB SRAM (16 KB DMEM and 24 KB PMEM). Area consumption caused by ISA extension are 7862 gates, which is about 4% of the total area. The power consumption is 58 mW. Figure 9 is the layout of multimedia surveillance chip. At the bottom are 2 DSP clusters, each DSP cluster consisting of a CDSP. The chip can output at least 1080P or 4 channel D1 video format in real-time. Figure 10 is the photo of the chip.

## 6. Conclusions

This paper proposes our approach and experiences for designing a platform based on CDSP, a clustered VLIW DSP with ISA extension for cryptographic algorithms. CDSP is designed to target the solving of privacy and security issues in multimedia surveillance system. CDSP has 11 pipeline stages, making it achieve high frequency of 360 MHz. CDSP has six function units, and can run up to six instructions in one cycle, largely enhancing the calculation density. The architecture of CDSP is advantageous in exploiting instruction level parallelism and achieving better performance.

ISA of CDSP consists of both general instructions and dedicated instructions. In this work, we presented our experience for design dedicated instructions for DES and AES algorithms. The result shows that those dedicated designed instructions can significantly improve the performance, and reduce software code size. Since cryptographic algorithms usually consist of special complex computation-intensive operations, making software solution yield poor throughput, according to our results, adding dedicated instructions is a good choice to improve performance, and is more convenient compared with the co-processor scheme.

Many common cryptographic algorithms are already implemented in CDSP, including block cipher algorithms, hash functions and public key cryptographic algorithms. Using our approach, new data encryption algorithms could be easily implemented on CDSP platform, making CDSP a practical solution for building, establishing a complete network security system.

## Figures and Tables

**Table 1:** Assembly code for DES Algorithm.

Cycle	Instruction
1	XD, G3:G2 = LD.D (X0++)
2	XD, G3:G2 = LD.D (X0++)
3	XA, G1:G0 = DESIP (G1:G0)
	XD, G3:G2 = LD.D (X0++)
4	XA, G1:G0 = DESRND (G1:G0, G3:G2)
5	XA, G1:G0 = DESRND (G1:G0, G3:G2)
…	… …
20	XA, G1:G0 = DESFP (G1:G0)

**Table 2:** Code size comparison for DES (instruction number).

Algorithm	Dedicated Inst.	General Inst.
DES_encryption	56	∼500
DES_decryption	57	∼500
3DES_encryption	155	∼500
3DES_decryption	158	∼500

**Table 3:** Assembly code for AES Algorithm.

1	XD, X1:X0 = LD.D (G7)
	YD, Y1:Y0 = LD.D (G7++[#2])
2	NOP
3	NOP
4	XA, G0 = XOR (G0, X0)
	XM, G1 = XOR (G1, X1)
	XA, G2 = XOR (G2, Y0)
	XM, G3 = XOR (G3, Y1)
	XA, G1:G0 = AESSHF (G1:G0, G3:G2)
5	YA, G3:G2 = AESSHF (G3:G2, G1:G0)
Loop	XD, X1:X0 = LD.D (G7)
	YD, Y1:Y0 = LD.D (G7++[#2])
6	XA, G0 = AESSUBMIX (G0)
	YA, G1 = AESSUBMIX (G1)
7	XA, G2 = AESSUBMIX (G2)
	YA, G3 = AESSUBMIX (G3)
8	XA, G0 = XOR (G0, X0)
	XM, G1 = XOR (G1, X1)
9	YA, G2 = XOR (G2, Y0)
End	YM, G3 = XOR (G3, Y1)

**Table 4:** Code size comparison for AES (instruction number).

Algorithm	Dedicated Inst.	General Inst.
AES_encryption	65	∼300
AES_decryption	63	∼300

**Table 5:** Block cipher performance (360 MHz).

		No Mode	ECB	CBC	CFB/OFB	CTR
DES	cycles	21	41	43	43	NA
	throughput (Mbps)	1097	562.0	535.8	535.8	NA
3DES	cycles	53	73	75	75	NA
	throughput (Mbps)	434.7	315.6	307.2	307.2	NA
AES	cycles	59	71	73	73	75
	throughput (Mbps)	781.0	649.0	631.2	631.2	614.4
IDEA	cycles	57	69	71	71	NA
	throughput (Mbps)	404.2	333.9	324.5	324.5	NA

**Table 6:** AES128 performance comparison with general purpose processors (Cycles/Byte).

Ref. [19]	ARM7	104.69
Ref. [19]	ARM9	86.5
Ours	CDSP	4.56

**Table 7:** AES128 performance comparison with other ISA extension methods (Cycles/Byte).

		ECB	CBC	CTR
Ref. [17]	Power8	-	5.53	-
Ours	CDSP	4.44	4.56	4.49
Ref. [16]	Intel	1.28	4.15	1.38

**Table 8:** AES128 performance comparison with other DSP implementations (Cycles/Byte).

Ref. [8]	TMS320C6201	14.25
Ref. [14]	Jazz DSP	10.56
Ours	CDSP	4.56

**Table 9:** Hash function performance (360 MHz).

	Cycles	Time (ns)	Throughput (Mbps)
MD5	325	902.8	567
SHA-1	316	877.8	583

**Table 10:** RSA and ECC performance.

	Cycles (MUL)	MUL Operation	Cycles	Time (ms)	Tran./s
RSA1024	2840	1536	4,367,000	12.13	82.0
RSA1024(CRT)	815	1536	1,223,000	3.397	294.4
RSA2048	10,512	3072	32,313,800	89.76	11.1
RSA1024(CRT)	2840	3072	8,751,000	24.31	41.1
ECC192	163	1920M + 1536S	742,200	2.062	485.0
ECC256	301	2560M + 2048S	1,651,000	4.586	218.1

**Table 11:** RSA performance comparison with other DSP implementations (ms).

		RSA1024	RSA2048
Ref. [9]	TMS320C6201	11.7	84.6
Ours	CDSP	12.13	89.76
